# Notices, Etc.

**Published:** 1861-11

**Authors:** 


### Notices, etc.

---

### MUSICAL INCIDENT.

A well-dressed gentleman called the other day to sell some patriotic music adapted to the Piano, and seeing the instrument open, he offered to play one of the pieces, and thus give an idea of its beauty. The instant he touched the keys, he turned round, and with a mingled expression of intelligence and gratification, exclaimed, "This must be one of Worcester's;" and so it was. But the story does not end here. The chords were so perfect, and the tones so clear and distinct, that he seemed to forget himself, and played half a dozen pieces, one running into the other, so that we began to fear he was like the man with the cork leg, which worked so well he couldn't stop. Why this maker's instruments maintain their superiority was lately explained in our office by one of the very first piano-workmen in the city—not in Mr. W.'s employ. "There is no shop known to me where such extraordinary pains are taken to make every part of the instrument as perfect as possible as in the old and extensive establishment of Horatio Worcester in Fourteenth street."

### EDUCATION.

Not many editors advertise a man without being asked; but our notices are intended to benefit our subscribers, by placing before them items of intelligence which will either promote their comfort or save money, and oftentimes both at once. Painfully earnest inquiries are made, from time to time, by parents from the country, in limited circumstances, as to where their children could obtain a good education at a moderate cost. The private schools of Mr. Abbott, in Fifth Avenue, and of Miss Hains, on Gramercy Park, are of a high order, and need no commendation. But only a favored few can afford to pay from four to seven hundred dollars a year for the education of a single child. It is a welcome task, under these circumstances, to notice an advertisement in that excellent and favorite family paper, the *Home Journal*, ($2 a year,) of the Grammar School of Madison University, at Hamilton, N. Y. Classical, to prepare for college; and English, to prepare for business. Three terms, opening October 19th, January 17th, and May 23d. Ninety-five dollars pays for board, tuition, room-rent, washing, lodging, and incidentals, for one year, not including, we presume, the recesses. It is most ravishingly refreshing to know that there is one educational establishment in the universe where there are no "extras." Why, nine tenths of the private schools have a list of "incidentals," in their bills a mile long, "more or less." We personally know nothing about the merits of the above school, but it is doubtless quite as good as others. One thing looks well; it don't brag. It don't parade an interminable column of honorable and reverend referees, three fourths of whom have given their sign-manual merely to be accommodating, or for the purpose of being found in good company.

One of the best private boy-schools in New-York is kept by Mrs. Dr. Steele, at 70 Irving Place. Those who want a thorough teacher for their little girls will find such at 58 East Twenty-fifth street, in the person of Mrs. McMillin, the wife of a returned and disabled foreign missionary.

### COMMENDATORY.

Says the Baltimore *True Union* of the 12th of September: "We have had occasion frequently to commend the good common-sense and judicious counsels of Dr. W. W. Hall, of New-York, contained in this *Journal*. The September number is not an exception. Its advice is invaluable to clergymen and heads of families. By following it we doubt not many times the cost of the *Journal* (only $1 per annum) would be saved from the apothecary's bill in a year. By the way, the Doctor gives us a rap for copying extracts from his paper without credit. We need not assure him that we have never done so knowingly. We have doubtless cut the extracts complained of from *other papers*, where they were first inserted without credit. We owe Dr. Hall too much for his good advice many years ago to do him wrong. When we had been laid aside from the pulpit for years by a bronchial affection, and were rapidly sinking into a state of confirmed *invalidism*, we consulted him, and are largely indebted to his advice for the improved health we have since enjoyed, and for more than one hundred and fifty sermons we have been permitted to preach since we had abandoned the pulpit, as we then feared, forever."

### MATRIMONIAL.

We have a hope and a wish. The hope is, that the writer in the *Home Journal*, on "Matrimonial Infelicities," has a few more articles of the same sort left. And the wish, that they may be embodied in a dollar volume. They are so full of human nature, so full of fun at the expense of water-weak husbands, there is no doubt of its making a good "Doctor-Book." Besides the laughter and the mirth generated, there is a spice of deep comfort in these papers. Every paragraph, almost, goes right home to the consciousness of the individual, giving "aid and comfort" in the direction indicated by that spiteful old French curmudgeon, who first enunciated the sentiment, that the misfortunes of our best friends were not without a mite of pleasurableness to the very best of us. To make it apply: Suppose a lady has a husband who is everlastingly growling; there is some pleasure in the intelligence that some other woman has a similar contemptibility. "Supposing," on the other hand, the husband has ordinary prudence; has a wholesome fear of debts, difficulties, and due-bills, and spies hobgoblins dire in an unruled household—hence to be master of "the situation," exercises a quiet, steady, and firm control over the whole domain, exacting regularity, system, promptitude, economies, and healthful observances, requiring in all cases that actual possession must precede disbursements—it is not a wonder, that when once in a decade a woman is found to be the owner of such a husband, number one feels "glad of it," on the ground that "misery loves company," albeit that said misery is in Betty Martin's eye. There is one difficulty in reading the Matrimonial Arenas, each party will look over the other side of the fence instead of at itself. The wife exclaims, when the husband gets a "dig," that's "him" exactly; and the husband, when the "poor, oppressed, suffering" wife comes off second best, asseverates, with an almost savage delight, "That's my wife to a T. Thank my stars, I'm not alone in my misfortunes." We will venture the assertion that no series of articles domestic, since the Caudle Lectures and Sparrowgrass Papers, have been read with such a peculiar gusto as the "Matrimonial Infelicities," in the *Home Journal* of Morris & Willis—two dollars a year only. Why, one of the Infelicities is worth two dollars; for example, when Hubby forgot to kiss his wife until he had taken his seat in the omnibus, and then went back and got a dozen.

### PACKING FRUIT.

Kidd, the gardener of the Marquis of Broadalbane, who sends fruits and flowers from the garden, near Hampton Court, England, to the Highland residence of the Marquis, subject to five hundred miles' carriage, is so successful in packing, that he can send fully ripe peaches "without losing a fruit," and bouquets, that when received will be as fresh as when first picked. A layer of bran is put at the bottom of a box or cask; then each bunch of grapes is held by the hand over the center of a sheet of paper; the four corners of the paper are brought up to the stalk and nicely secured; then laid on its side in the box, and so on until the first layer is finished. Then fill the whole over with bran, and give the box a gentle shake as you proceed. Begin the second layer as the first, and so on, until the box is completed. Thus, with neat hands, the bloom is preserved, and may be sent to any distance. He has invariably packed from sixty to eighty bunches of grapes, and fifty or sixty dozen of peaches or apricots, in one box, and received letters from employers, to say that they had arrived as safe as if they had been taken from the trees that morning.

